# Effects of Short-Term Straw Return and Manure Fertilization on Soil Microorganisms and Soybean Yield in Parent Material of Degraded Black Soil in Northeast China

**DOI:** 10.3390/microorganisms13051137

**Published:** 2025-05-15

**Authors:** Jiahua Ding, Zhao Li, Jiali Wu, Dalong Ma, Qiang Chen, Jianye Li

**Affiliations:** 1 College of Geographical Science, Harbin Normal University, Harbin 150025, China; dingjiahua@stu.hrbnu.edu.cn (J.D.); lizhao@hrbnu.edu.cn (Z.L.); 2024301047@stu.hrbnu.edu.cn (J.W.); madalong728@163.com (D.M.); 2 Key Laboratory of Mollisols Agroecology, Northeast Institute of Geography and Agroecology, Chinese Academy of Sciences, Harbin 150081, China; lijianye@iga.ac.cn

**Keywords:** degraded black soil, straw return, manure fertilization, microbial diversity, soybean yield

## Abstract

Soil erosion has caused the loss of black soil and exposed the soil parent material in the cultivated layer of sloping farmland in Northeast China. Straw return (STR) and manure fertilization (MF) are critical measures to improve soil quality and crop yield. However, the effect of STR and MF on the soil properties of the parent material remains unclear. We conducted a 1-year pot experiment in the field using the soil parent material of degraded black soil to evaluate the effects of STR and MF on soil nutrients, microbial community, and soybean yield. We analyzed these effects using two treatments (STR and MF) in three soybean growth stages (seedling, flowering, and maturity) and a control group (CK). The MF treatment had higher α and β diversity of soil microbial than the CK during all soybean growth stages. Similarly, STR had higher soil microbial α diversity at the maturity stage and lower diversity at the seedling stage. Co-occurrence network analysis suggested that STR and MF increased the proportion of positively correlated edges in soil bacterial and fungal networks compared to the CK. Notably, the treatments enriched beneficial taxa, such as *Schizothecium* (fungi) and *Massilia* (bacteria), which are associated with organic matter decomposition and nitrogen cycling. STR and MF significantly improved soil organic matter, total nitrogen, and carbon-nitrogen ratio (*p* < 0.05). Structural equation modeling (SEM) revealed that STR and MF directly increased soybean yield. This effect was primarily mediated by the significantly higher soil organic matter, total carbon, total nitrogen, and carbon-to-nitrogen ratio in the treatments than in the CK (*p* < 0.05). In summary, STR and MF improved soil fertility and soil microbial community diversity of degraded black soil. This study provides scientific methods to improve the fertility of degraded black soil and increase soybean production in the short term.

## 1. Introduction

The black soil region of Northeast China is an important commercial grain production base and is regarded as “the hometown of soybeans” [1]. However, inappropriate land development and utilization have caused topsoil loss of sloping farmland at rates of 0.3–1 cm annually. Consequently, the average soil layer thickness has decreased from 60–80 cm in the 1950s to 20–30 cm at present [2,3]. Continued land degradation has caused the complete disappearance of the black soil layer in some areas of sloping farmland, exposing the soil parent material [4,5], which has less organic matter and a worse soil structure than the black soil, resulting in reduced grain yields and threatening China’s food security [6,7]. These severely degraded areas still need to be used as arable land to protect the arable land minimum of China, so it is critical to rapidly restore the soil fertility of degraded black soils to maintain grain production in Northeast China. Meanwhile, Soybean is a relatively low-tilled crop, which can increase soil nitrogen content through nitrogen fixation and improve soil fertility [8]. In addition, previous studies found that short-term STR could increase soybean yields while reducing corn yields by 30% in this region [9].

The addition of organic materials (i.e., straw and manure fertilization) is crucial for improving soil quality and crop yield [10,11]. Numerous studies have shown that applying organic materials improved soil structure, water-holding capacity, and nutrient availability and content [12,13,14,15]. Moreover, soil microbes are crucial in maintaining soil quality in farmland ecosystems [16]. The application of organic material substantially improved soil microbial richness, diversity, and functional gene abundance [14,17]. Long-term (44-year) applications of MF increased the relative abundance of *Nitrospira* and *Gemmatimonas*, which were significantly positively correlated with soybean yield in Northeast China [18]. Meanwhile, the STR treatment provided nutrients for soil microorganisms, increased soil microbial diversity, and optimized microbial community structure [12,19,20]. Furthermore, improving soil organic matter, nutrient cycling, and carbon sequestration, and increasing soil fertility and crop yields [21,22,23].

The utilization of straw and manure fertilizers has become a critical priority for sustainable agriculture in the black soil region of Northeast China [14,24,25]. Several studies have demonstrated that the application of STR and MF can improve soil structure, increase soil nutrients, and maintain crop yields in this region [15,26,27]. However, few studies have analyzed the addition of straw and MF on soil quality and crop yields in the significantly eroded areas, i.e., regions where the soil parent material is exposed. We hypothesized that the application of organic materials can modulate microbial structure and function by improving soil fertility, thereby enhancing soybean productivity in the soil parent material of degraded black soil in Northeast China. Our objectives were to (1) evaluate the short-term effects of STR and manure fertilization (MF) on soil microbial community composition and abundance in degraded black soil, (2) examine the relationships between soil microbial communities, soil physicochemical properties, and crop yields, and (3) identify suitable practices for rapidly improving soil fertility and crop productivity. These results can provide a scientific basis for the sustainable utilization of arable land in Northeast China.

## 2. Materials and Methods

### 2.1. Experimental Site

The field experiment was conducted at the Hailun Monitoring and Research Station of Mollisol Erosion, Chinese Academy of Sciences (47°21′ N, 126°50′ E), in Hailun City, Heilongjiang Province. The mean annual air temperature is 1.5 °C. The ≥10 °C accumulated temperature ranges from 2400 to 2500 °C, and the mean annual sunshine duration ranges from 2600 to 2800 h. The area has a temperate continental monsoon climate with a mean annual precipitation of 530 mm, 65% of which falls from June to August. The frost-free period is approximately 120 days.

### 2.2. Experimental Design

**Figure 1:**
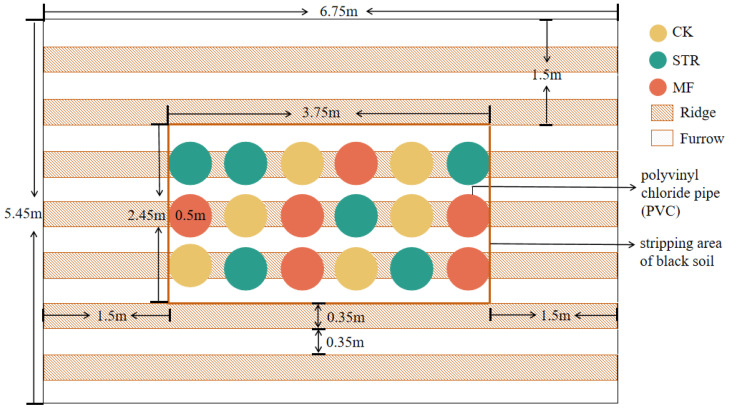
Experimental plot. CK: the control treatment without the additions of straw and manure fertilization; STR: Straw returning; MF: Manure fertilization.

Experiment plots were established in the autumn of 2022. We conducted pot experiments in the field (Figure 1). An experimental farmland area (3.75 m length × 2.45 m width × 0.45 m height) was selected, and the naturally degraded soil was removed. Polyvinyl chloride (PVC) pipes (0.5 m in diameter and 0.5 m in height) were placed into the experimental area to delineate farmland ridges. The bottoms of the PVC pipes were covered with nylon mesh (30 μm) to exclude roots and facilitate soil moisture movement [28]. The PVC pipes were filled with the air-dried parent material of the black soil (bulk density: 1.50 g/cm^3^) [29], which was passed through a 2 cm sieve to ensure homogeneity. The soil properties are listed in Table 1. To control boundary effects, the spatial interval between each plot was 20 cm, and the height and depth of the PVC pipes were 50 cm and 45 cm, respectively. This means that the PVC pipe was 5 cm higher than the surface ground, which was beneficial to prevent the plot from being mixed into the plot during the snowmelt and rainy periods. Meanwhile, the plastic films were covered in the plots to reduce the impact of wind erosion before sowing in spring. In addition, a 1.5-meter-wide buffer planted with soybeans was created around the perimeter of the experimental site to protect the site from external influences. The spaces between the PVC pipes were backfilled with the removed soil.

The field experiment plots included two treatments (STR and MF) and a control group. We used a randomized complete block design with six replications. The MF treatment was 1.5 t/hm^2^, with an average organic matter content of 47 g/kg. The STR treatment consisted of applying crushed maize straw at a rate of 10 t/hm^2^, with an average total organic carbon content (TC) of 44 g/kg and total nitrogen content (TN) of 4 g/kg. The manure fertilization and crushed corn straw were evenly incorporated into the 0–20 cm soil layer in autumn 2022. Ten soybeans were planted in each plot, combining the regional survey data, and the same field managements were applied to all the plots. Inorganic fertilizer was applied to the treatment and control plots based on the typical fertilizer amount in the area at 20.25 kg N/ha, 51.75 kg P/ha, and 15.00 kg K/ha to provide soil nitrogen, phosphorus, and potassium [9,30].

### 2.3. Soil Sampling and Measurement

Soil samples were collected at 0–20 cm soil depth during the soybean growth stages, sowing (May), flowering (July), and maturity (October). The mixed soil samples of the three sampling points were stored in sealed plastic bags. They were divided into two parts. One part was air-dried and passed through a 0.25 mm sieve to measure soil physicochemical properties. The other part was stored at −20 °C before analyzing soil microbial communities. Soil pH was determined using a potentiometer with a fixed ratio of 1:2.5 soil to water. Soil total carbon (TC) and total nitrogen (TN) were measured using an elemental analyzer (Vario EL, Langenselbold, Germany). Soil temperature and soil moisture were measured using a FieldScout TDR 350 (Aurora, IL, USA).

### 2.4. Soil DNA Extraction and High-Throughput Sequencing

Soil DNA was extracted from 0.5 g of a fresh soil sample using the PowerSoil DNA Isolation Kit (Mo Bio, Carlsbad, CA, USA). The quantity and quality of the extracted DNA were assessed using a NanoDrop 2000 spectrophotometer (Thermo Fisher Scientific, Wilmington, DE, USA) and a Qubit 3.0 Fluorometer (Thermo Fisher Scientific, Wilmington, DE, USA), respectively. The bacterial 16S rRNA gene was amplified with primer pair 338F (5′-ACTCCTACGGAGGCAGCAG-3′) and 806R (5′-GGACTACHVGGGTWTCTAAT-3′) [31]. The fungal internal transcribed spacer (ITS) regions were amplified with primers TS1F (5′-GGAAGTAAAAGTCGTAACAAGG-3′) and ITS2R (5′-GCTGCGTTCTTCATCGATGC-3′) [32]. The polymerase chain reaction (PCR) reaction mixture consisted of 4 μL of 5× FastPfu Buffer, 2 μL of dNTPs (2 mmol/L), 0.8 μL each of forward and reverse primers (5 μmol/L), 2 μL of DNA template, 0.4 μL of Taq polymerase (5 U/μL), and ddH2O for a final volume of 20 μL [33]. The PCR cycle included initial denaturation at 95 °C for 5 min, followed by 35 cycles of denaturation at 95 °C for 30 s, annealing at 58 °C for 30 s, and extension at 72 °C for 1 min. The PCR product was extracted from a 2% agarose gel and purified using the PCR Clean-Up Kit (YuHua, Shanghai, China). Paired-end sequenced on an Illumina Nextseq2000 platform (Illumina, San Diego, CA, USA). The sequencing and analysis of the soil samples were conducted using an Illumina Miseq platform (Meiji Biotechnology Co., Ltd., Shanghai, China).

### 2.5. Statistical Analysis

The raw sequencing data were quality-filtered using Fastp (version 0.19.6) to remove low-quality base sequences. Sequence clustering was performed using Uparse (version 11.0) with a 97% similarity threshold for operational taxonomic unit (OTU) designation. After rarefaction analysis based on the minimum sequence count across samples, 34,740 high-quality sequences were retained and clustered into 3234 OTUs. Taxonomic annotation was performed using the Ribosomal Database Project’s (RDP) Bayesian classification algorithm and the Silva SSU128 16S rRNA and fungal 18S rRNA reference databases. Alpha diversity was calculated using Mothur (version 1.30.2). The original data were processed by EXCEL 2011 software and then were normalized by using descriptive Statistics from the SPSS 26.0 software (IBM Corp., Armonk, NY, USA): One-way ANOVA and Duncan’s multiple-range test were used to determine statistically significant differences in the soil physiochemical properties, microbial diversity and crop yields between treatments. In this study, the Kolmogorov–Smirnov statistical test for the analysis of homogeneity of variance was conducted.

The beta diversity pattern was assessed using principal coordinate analysis (PCoA) and analysis of similarities (ANOSIM) to identify significant microbial community differences between treatments. The co-occurrence network was constructed by using the top 150 OTUs with strong correlations (Spearman’s |r| > 0.7, *p* < 0.01), and the results were visualized using Gephi 0.10.0. Structural equation modeling (SEM) was used to analyze the direct and indirect effects of different organic materials on the degraded black soil’s properties, soil microorganisms, and crop yields.

## 3. Results

### 3.1. Soil Physical and Chemical Properties

MF increased soil pH, TC, TN, and soil organic matter (SOM) during all the soybean growth periods. The SOM content was significantly higher (*p* < 0.05) in the MF treatment in the seeding (23.96%), flowering (43.4%), and maturity (40.81%) stages than in the CK (Table 2). The STR treatment increased the TC, TN, and SOM contents. The average values of the TC, TN, and SOM in the MF treatment were 66.28%, 36.17%, and 45.79% higher in the MF treatment than in the CK (*p* < 0.05). Similarly, the average values of the TC, TN, and SOM in the STR treatment were 53.67%, 21.28, and 28.47% higher in the STR treatment than in the CK.

Moreover, a significantly higher carbon-nitrogen ratio (C/N ratio) was observed in the STR and MF treatments than in the CK in the flowering and maturity stages. No significant differences in soil moisture and soil temperature occurred in the treatments during the soybean growth season. In addition, the TC, TN, and SOM in the STR and MF treatments were higher in the flowering and maturity stages than in the seedling stage.

### 3.2. Bacterial and Fungal Diversities

**Figure 2:**
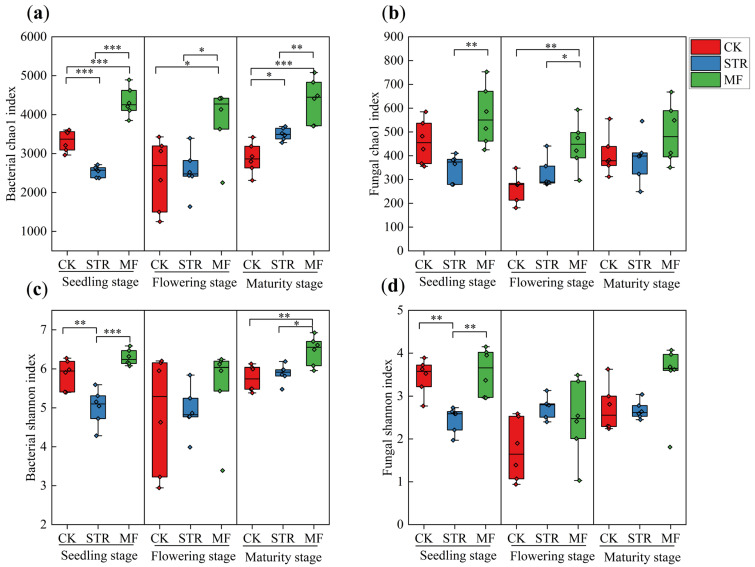
Effect of the application of straw return (STR) and manure fertilization (MF) on the Alpha diversity index of bacteria (**a**,**c**) and fungi (**b**,**d**) in different periods. Note: *, ** and *** represented at *p* < 0.05, *p* < 0.01 and *p* < 0.001, respectively.

The alpha diversity of soil bacteria (the Chao1 and Shannon indices) was significantly lower in the STR treatment than the CK in the seeding stage (*p* < 0.05) (Figure 2a,c). In contrast, the Chao1 index was significantly higher in STR than in the CK in the maturity stage (*p* < 0.05). No significant difference in the Shannon index was found between STR and CK in the flowering and maturity stages. The Chao1 and Shannon indices were higher in the MF treatment than in the CK, especially in the seeding and maturity stages (*p* < 0.05) (Figure 2a,c).

STR significantly reduced soil fungal alpha diversity in the seeding stage, whereas no significant differences were observed in the flowering and maturity stages (Figure 2b,d). No significant differences in the alpha diversity of soil fungi were observed between MF and CK during the growing season (Figure 2b,d).

### 3.3. β Diversity of Bacterial and Fungal Communities Under the Addition of Different Organic Materials

**Figure 3:**
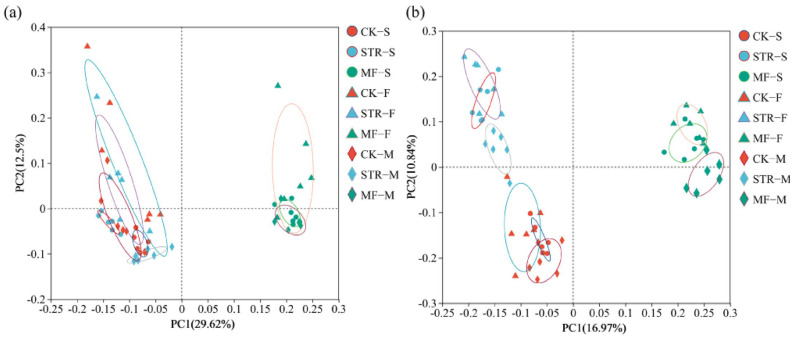
Principal coordinate analysis of soil bacterial (**a**) and fungal (**b**) communities under the addition of different organic materials (PCoA). Note: CK: the control treatment without the additions of straw and manure fertilization; STR: Straw returning; MF: Manure fertilization; S, F, M, represent the seedling, flowering, and maturity stages, respectively.

The PCoA revealed significant differences in bacterial and fungal communities among the treatments (Figure 3). PC1 and PC2 accounted for 29.62% and 12.50% of the total variance in bacterial community structure (Figure 3a) and 16.97% and 10.84% of the variance in fungal community structure, respectively (Figure 3b). The bacterial and fungal communities of the CK group and the STR treatment were widely separated from those of the MF treatment along the PC1 axis, suggesting similar microbial community structures in the CK and STR. Thus, we classified them into CK + STR and MF groups. Significant differences in the soil bacterial community were observed between samples collected at different times (seedling stages and flowering stages). However, the fungal communities at the seeding and maturity stages clustered together and were distinct from those at the flowering stage, indicating that the crop growth period significantly affected soil microbial community structure.

### 3.4. Composition of Bacterial and Fungal Communities in Different Soybean Growth Stages

**Figure 4:**
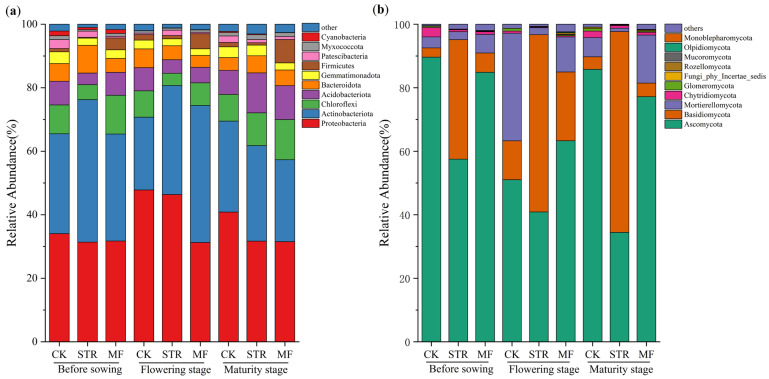
Effect of the application of straw return and manure fertilization on the top 10 species phylum relative abundance of soil bacterial community (**a**) and fungal community (**b**).

The bacterial community was analyzed at multiple taxonomic levels. High-throughput sequencing identified 32 phyla, 89 classes, 209 orders, 331 families, and 666 genera. At the phylum level, the dominant bacterial phyla in all treatments exhibited relative consistency during the crop growth season, including *Proteobacteria*, *Actinobacteriota*, *Chloroflexi*, *Acidobacteriota*, and *Bacteroidota*. These accounted for 86.19–93.35% of the total bacterial abundance (Figure 4a). The abundance of *Actinobacteriota* was higher in the STR and MF treatments than in the CK, while the relative abundance of *Proteobacteria* was lower. Moreover, the relative abundance of *Chloroflexi* and *Acidobacteriota* in STR was higher in the maturity stage than in the seedling and flowering stages. The relative abundance of *Firmicutes* in the MF treatment increased significantly during the crop growth period (*p* < 0.05).

**Figure 5:**
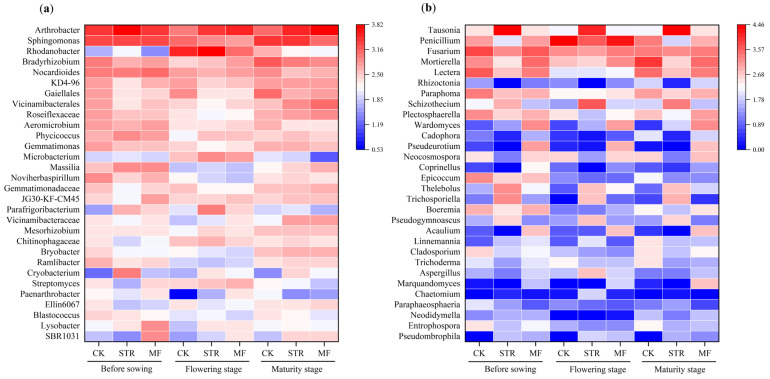
Effect of adding different organic materials on the genus-level composition of soil bacterial community (**a**) and fungal community (**b**).

The MF significantly decreased the abundance of *Gemmatimonas* and *Sphingomonas* (Figure 5a). In contrast, STR decreased the abundance of *Gemmatimonas* but enhanced the abundance of *Arthrobacter* during the soybean growing stages. The abundance of *Rhodanobacteria* in the three treatments was highest in the flowering stage.

As shown in Figure 4b, 14 phyla, 42 classes, 86 orders, 166 families, and 304 genera of fungi were detected in the three treatments during the crop growth season. The dominant phyla included *Ascomycota*, *Basidiomycota*, and *Mortierellycota*, accounting for 96.32–98.43% of the relative abundance in all treatments (Figure 4b). STR significantly reduced the relative abundance of *Ascomycota* and *Mortierellycota* and increased the relative abundance of *Basidiomycota* (*p* < 0.05). The MF treatment increased the relative abundance of *Basidiomycota* at all growth stages and reduced the relative abundance of *Ascomycota* at the seeding and maturity stages. The changes in the abundance of *Mortierellycota* were opposite to those of *Ascomycota* at various stages in the MF treatment.

At the genus level, STR significantly increased the relative abundance of *Tausonia*, *Schizothecium*, *Thelebolus*, *Pseudogymnocus*, and *Trichosporiella* and decreased the abundance of *Penicillium*, *Mortierella*, *Fusarium*, *Lectera*, and *Boeremia* (*p* < 0.05). The abundance of *Waromyces*, *Acaulium*, and *Pseudourotium* was significantly higher in the MF treatment than in the CK (*p* < 0.05) (Figure 5b).

### 3.5. Effects of Different Organic Materials on Soil Microbial Co-Occurrence Network

**Figure 6:**
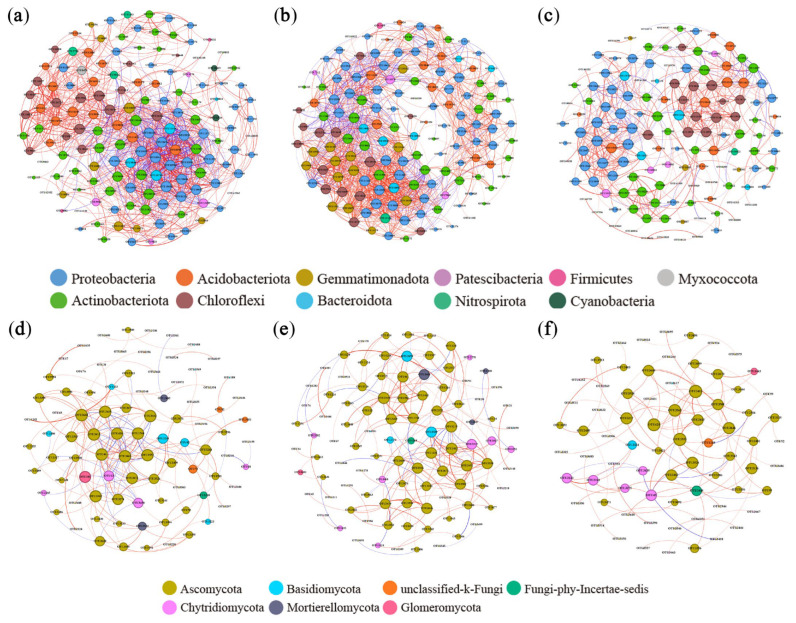
Co-occurrence network of soil bacteria and fungi under the application of straw return and manure fertilization: (**a**) Bacterial CK; (**b**) Bacterial STR; (**c**) Bacterial MF; (**d**) Fungal CK; (**e**) Fungal STR; (**f**) Fungal MF. Nodes represented operational taxonomic units (OTUs) color-coded by different phyla and scaled proportionally to the number of connections (node degrees). Edges represented correlations between OTUs and were scaled proportionally to the weight of the edge. Connection lines at r > 0.7 (positive correlation, red) or r < −0.7 (negative correlation, blue) and *p* < 0.01.

Co-occurrence networks of soil bacteria and fungi were constructed to analyze microbial community interactions in different organic material treatments (Figure 6). Higher proportions of positive correlations occurred between bacteria and fungi in the STR and MF treatments. The results demonstrate enhanced mutual synergistic interactions, metabolic efficiency, and environmental adaptability in microbial communities. The analysis of network topological parameters (Table 3) revealed that the graph density and number of edges were higher in the STR treatment than in the CK, indicating greater network complexity. The MF treatment had lower network complexity. The modularity coefficients were higher in STR and MF than in CK.

We identified the keystone species by calculating the degree centrality, closeness centrality, and betweenness centrality in different treatments. The keystone species in the co-occurrence network of bacterial communities were *Chujaibacter* and *Ramlibacter* in the CK, *Vicinamibacteraceae* and *norank_o__IMCC26256* in the STR treatment, and *Sphingomonas* and *Massilia* in the MF treatment. In the fungal communities, the keystone species were *Penicillium* and *Boeremia* in the CK, *Aspergillus* and *Schizothecium* in the STR treatment, and *Boeremia* and *Lectera* in the MF treatment.

### 3.6. Soybean Yields

**Figure 7:**
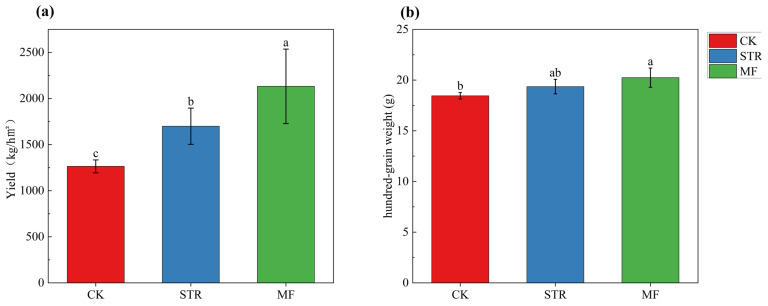
Soybean yields (**a**) and hundred-grain weight (**b**) in the straw returning (STR) and manure fertilization (MF). Notes: Different lower-case letters represent the significant differences between the treatments (*p* < 0.05).

The STR and MF treatments significantly increased (*p* < 0.05) soybean yield by 34.52% and 68.8%, respectively (Figure 7). The hundred-grain weight of soybean was significantly higher in MF than in CK (*p* < 0.05), whereas no significant difference was found between the STR treatment and the CK.

### 3.7. Relationship Between Soil Properties and Soybean Yield

**Figure 8:**
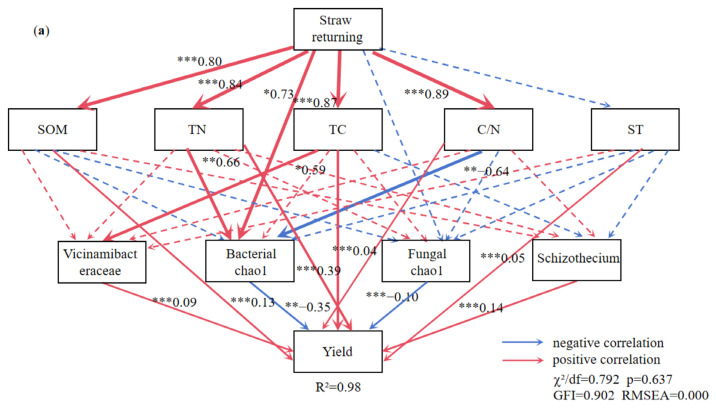

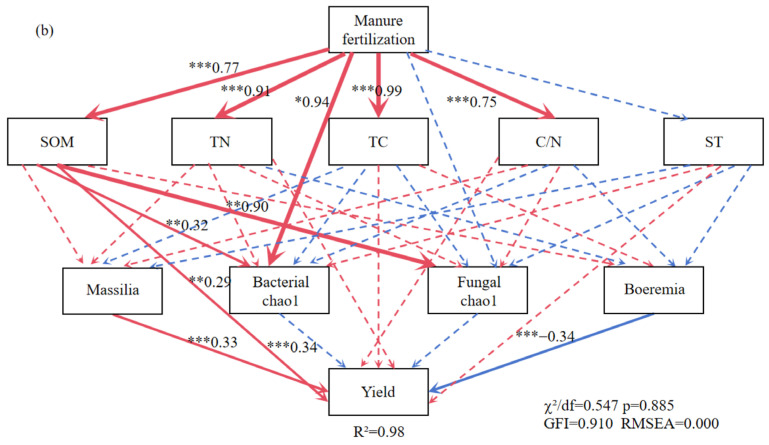
The structural equation model reveals the relationship between straw returning (**a**), manure fertilization (**b**). Note: ST: soil temperature; TC: soil total carbon; TN: soil total nitrogen; C/N: soil carbon-nitrogen ratio; SOM: soil organic matter. The red arrow indicates positive correlation and the blue arrow indicates negative correlation. Solid lines indicate significant correlation, dotted lines indicate insignificant, *, ** and *** represent *p* < 0.05, *p* < 0.01 and *p* < 0.001, respectively, and the width of the arrow is proportional to the intensity of the path coefficient.

The TC, TN, SOM, C/N ratio, and the Chao1 index of soil bacterial diversity exhibited significant positive correlations with the STR treatment (Figure 8a). The SOM, TN, ST, C/N ratio, and the abundance of *Schizothecium* and *Vicinamibacteraceae* were significantly positively correlated, and the Chao1 index of soil bacterial and fungal diversity was significantly negatively correlated with the soybean yield (Figure 8a).

**Figure 9:**
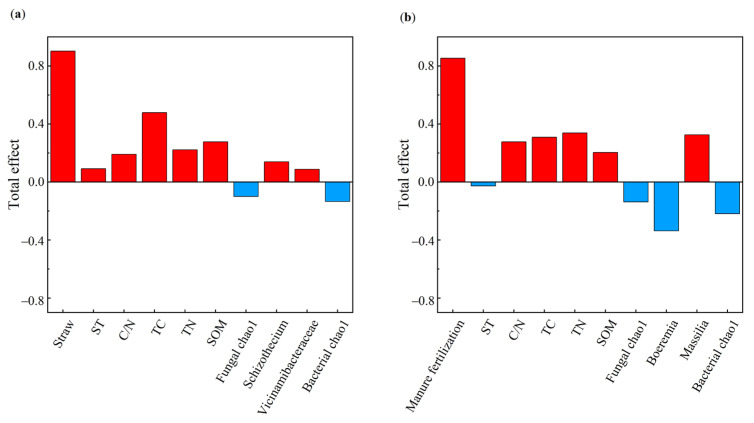
Soil physical and chemical properties and microbial properties, and crop yield. (**a**,**b**) are the total impact of different variables on yield. Note: ST: soil temperature; TC: soil total carbon; TN: soil total nitrogen; C/N: soil carbon-nitrogen ratio; SOM: soil organic matter. The red bar represents the positive effect and the blue bar represents negative effect, respectively.

The MF treatment was positively related to SOM, TN, TC, and the C/N ratio (Figure 8b). The SOM was positively correlated with soybean yield and indirectly influenced soybean yield by moderating the Chao1 index of soil bacterial and fungal diversity. *Massilia* (a dominant bacterium in MF) was highly positively correlated with soybean yield. Figure 9 illustrates the effects of the indicators on soybean yield. Soil physicochemical properties had a larger influence on soybean yield than microbial factors in the STR treatments, whereas both factors had comparable effects in the MF treatment.

## 4. Discussion

### 4.1. Effects of Straw Return and Manure Fertilization on Soil Microbial Diversity

Soil microorganisms are key indicators of soil quality and ecological functions. They are vital in maintaining farmland ecosystem sustainability [34]. A large number of studies have proved that long-term STR could increase α diversity of soil microorganisms [12,18,20]. However, the impact of short-term STR on soil microbial diversity remains debated. For instance, Liu et al. (2022) found that a 2-year STR experiment, across diverse regions of Jilin Province, revealed that α diversity of soil microbial communities exhibited significant decreases in specific areas while showing marked increases in others [35]. Wang et al. (2021) also demonstrated that different amount of straw retuning induced significant reductions in α diversity of soil fungal in short-term conditions [36]. These results are similar to our findings at the seedling stage. However, α diversity of soil microbial in STR treatment increased with time during the growing season. This was associated with the releases of substantial labile carbon in the initial stage of straw decomposition, creating nutrient-enriched conditions that selectively promote the proliferation of copiotrophic microorganisms (e.g., *Proteobacteria* and *Actinobacteria*), which thrive under high-resource availability [37]. Conversely, this process suppresses the activity of oligotrophic microorganisms adapted to nutrient-limited environments, thereby reducing bacterial diversity during the seedling stage [37,38]. Bacterial communities were dominant in the early-stage straw decomposition [39]. The intensive oxygen consumption creates hypoxic conditions that suppress aerobic fungal populations, reducing fungal diversity [36].

In contrast, MF had a more direct impact on soil nutrients, which showed MF treatment had higher α diversity of soil microbial than the CK during all soybean growth stages. Hence, MF treatment has stronger ecosystem functions and faster recovery from disturbances, contributing to ecological health and resilience. After the spring freeze–thaw cycles, some nutrients in the manure decomposed and were released into the soil, improving bacterial richness and diversity in the seedling stage. On the other hand, MF increased soil pH, alleviating soil acidification and reducing the inhibition of microbial growth [15,18,40]. This result aligns with the findings of Thompson (2017) [41], who used environmental samples and metadata from various regions worldwide and reported that soil microbial richness was the highest when the pH was near neutral, and the soil temperature was approximately 10 °C. Furthermore, the soil nutrient content in the STR treatment was higher in the flowering and maturity stages than in the seedling stage. This result can be attributed to rising temperatures enhancing straw decomposition and subsequent nutrient release [42]. Consequently, the soil nutrient content was higher in the STR treatment than in the CK during the flowering and maturity stages.

We also found that soil nutrients in the MF treatment increased during the crop growth season (Table 2). The MF treatment increased the richness and diversity of soil bacteria and fungi at all soybean growth stages. The primary reason is that the humified carbon in the manure is directly absorbed and utilized by crops, increasing the soil C/N ratio and stimulating the priming effect (PE) [43]. PE refers to a strong and short-term change in the turnover of soil organic matter caused by the supply of available fresh organic matter [44]. Various studies have pointed out that the PE significantly influences soil microbial communities, with its intensity primarily determined by the C/N ratio [44,45]. The high soil carbon-to-nutrient ratios induced by organic matter inputs promoted microbial nitrogen mineralization and drove a positive priming effect (PE) [45,46]. Another reason may be that MF treatment can significantly increase DOC and MBC in the soil’s labile organic carbon, which has a significant positive correlation with microbial growth [40].

### 4.2. Effects of Straw Return and Manure Fertilization on Soil Microbial Community Structure

The *Acidobacteria* are oligotrophic. The relative abundance of *Acidobacteria* in the seeding and flowering stage was lower in the STR and MF treatments than in the CK (Figure 4a). This result was due to the better adaptation of *Acidobacteria* to low-carbon environments [47]. These findings were similar to those of Zhang et al. (2021) [40], who observed that the application of a chemical fertilizer combined with STR and MF significantly reduced the relative abundance of *Acidobacteria* compared to no fertilizer treatment. Some studies found that the soil pH and carbon-to-nitrogen ratio substantially affected *Acidobacteria* [48,49]. In contrast, Liu et al. (2014) performed high-throughput sequencing and observed that the composition of *Acidobacteria* was not related to soil pH in the black soil area of Northeast China [49,50]. This result aligns with our findings; therefore, the significant increase in the relative abundance of *Acidobacteria* in the STR and MF treatments during the maturity stage was attributed to the C/N ratio. Moreover, Lauber et al. (2008) reported that the relative abundance of *Acidobacteria* was significantly positively correlated with the C/N ratio [51]. In this study, the relative abundance of *Actinobacteriota* in the STR and MF treatments increased during the seeding and flowering stages (Figure 4a). The *Actinobacteriota* are r-strategists with fast growth and require high availability of soil nutrients [52]. The relative abundance of *Firmicutes* was significantly higher in the MF treatment than in the CK in all growth stages, which was consistent with the findings of Ma et al. (2022) [52]. The relative abundance of *Firmicutes* was positively correlated with soil pH, TN, and other physicochemical properties [53]. In this study, the MF treatment increased soil nutrient content and PH. Some studies have found that *Firmicutes* were one of the dominant bacterial phyla in organic fertilizer treatment, and their relative abundance was positively correlated with SOM and crop yield [54,55,56].

At the genus level, the abundance of *Gemmatimonas* in the STR and MF treatments decreased during the crop growth stages, suggesting it was more adapted to oligotrophic environments [57]. This finding was also reported in other studies that observed a lower relative abundance of *Gemmatimonas* in STR than in non-STR treatments [12,20]. Despite *Gemmatimonas* belonging to the oligotrophic bacterium, soil denitrification exhibited a positive correlation with *Gemmatimonas* abundance. This suggests that the reduced populations of this bacterial group may decrease denitrification activity, potentially mitigating nitrogen loss while enhancing nitrate retention in soils [58]. We observed a lower abundance of *Sphingomonas* during the soybean growth period in the MF treatment than in the CK. Fang et al. (2024) obtained similar results [18]. The *Sphingomonas* is an oligotrophic bacterium that thrives in low-nutrient environments [59]. The MF treatment increased soil nutrient levels over time, reducing the abundance of *Sphingomonas*.

In the soil fungal communities, STR significantly increased the relative abundance of *Basidiomycota* at all crop growth stages, consistent with the findings of Zhang et al. (2021) [60]. *Basidiomycota* produce various peroxidases for lignin oxidation, which increases the lignin content and the decomposition rate of crop straw [57]. Therefore, the higher abundance of *Basidiomycota* significantly enhanced soil organic carbon accumulation capacity, and it was also a key factor in assessing land use effectiveness on maximizing carbon sequestration efficiency [61]. Song et al. (2022) found that the relative abundance of potential soil pathogens (*Fusarium*, *Lectera*, and *Boeremia*) was significantly positively correlated with *Ascomycota* and negatively correlated with *Basidiomycota* [62]. We observed similar results. Furthermore, the abundance of *Boeremia* and *Fusarium* significantly increased under continuous soybean monoculture, which was strongly associated with the elevated incidence of soybean root rot disease [63].

The STR treatment significantly increased the relative abundance of *Tausonia* at the genus level during all crop periods (*p* < 0.05). *Tausonia* is a well-documented saprophytic *ascomycete* crucial for organic matter degradation. It was associated with increased crop yields [64]. The relative abundance of *Fusarium* was lower in STR than in CK, which is consistent with the results reported by Tang et al. (2020) [65]. Some studies found that *Fusarium* species cause wilting and root rot diseases [62]. However, the STR treatment increased the relative abundance of inhibitory bacteria, such as *Pseudogymnoascus* and *Schizothecium*, reducing the relative abundance of *Fusarium* [66]. Moreover, the MF treatment significantly increased the abundance of *Acaulium* during all crop growth stages (*p* < 0.05). He et al. (2022) observed that *Acaulium* was the key microbial genus responsible for the degradation of cow dung and straw, increasing total sugar degradation [67].

### 4.3. Effects of Straw Return and Manure Fertilization on Key Species and Microbial Networks

The STR treatment increased the number of edges, average degree, and average clustering coefficient of the co-occurrence network of bacteria and fungi, indicating an increase in network complexity. As a result, closer interactions occurred among co-occurring species networks. The complexity of microbial ecological networks can directly reflect the stability and interactions of the ecosystem, such as positive (e.g., commensalism) and negative (e.g., competition) [68,69]. The nutrients and complex organic matter in straw need to be decomposed synergistically by multiple soil microorganisms, which leads to higher connectivity and complexity of the network [70]. Numerous studies have shown that long-term or short-term STR increases the soil microbial network complexity [18,35,71]. Notably, the MF treatment reduced the bacterial and fungal network complexity. This is mainly due to maintaining nutrient balance in the soil for MF treatment, thereby reducing the interactions between microbial communities for development [18,72,73].

### 4.4. Relationship Between Soil Physical and Chemical Properties, Microbial Communities, and Crop Yields

Long-term STR and MF could increase soil nutrient content, promote soil microbial activities, and increase grain production, resulting in direct economic benefits. In addition, it can also improve soil structure and reduce soil erosion to generate indirect economic benefits [52,74]. In contrast, the short-term application of organic materials may not be completely decomposed into available nutrients, resulting in the limitation of soil nutrients and soil microbial activities, and ultimately leading to lower yields [75].

As illustrated in Figure 7a, STR and MF significantly increased soybean yield (*p* < 0.05). This finding is consistent with the observation that the addition of organic material increased crop yields by altering soil microbial communities and improving soil properties [12,54]. Our study demonstrated that the STR and MF treatments significantly increased soybean yield by improving the C/N ratio and TN (*p* < 0.05). Therefore, the soil TN content is vital in determining crop yields in degraded black soil areas, which is in line with previous studies [12,18]. Furthermore, the increased crop yield was related to soil microbial communities, such as *Schizothecium* and *Massilia*, which processed organic materials, indirectly improving soybean yield [76,77].

The *Massilia* participates in the transformation of ammonia and nitrate, while significantly modulating NH_4_^+^-N content in soil ecosystems [78]. In addition, the *Massilia* is also a key species that affects the characteristics of soil nitrogen retention and supply. Several studies have confirmed that *Massilia* is critical for soil nitrogen fixation and utilization [79]. In this study, the relative abundance of *Massilia* in the MF treatment was positively correlated with soybean yield, and *Massilia* was identified as a keystone species. This is supported by the findings of Qiao et al. (2019), who observed that the application of bio-organic fertilizers significantly increased the relative abundance of *Massilia*, which was strongly correlated with increased crop yields [80].

The *Boeremia* was the key fungal genus in the MF treatment [80]. Its abundance was significantly negatively correlated with grain yield. Previous studies have identified *Boeremia* and *Lectera* as potential pathogenic genera in leguminous plants [62]. SOM provides all essential nutrients, including primary nutrients, sulfur, and micronutrients, for crop growth and yields, particularly in soils with low fertility [81]. This study found that SOM was significantly positively correlated with soybean yield because it alters the relative abundance of bacteria and fungi (*p* < 0.01). The *Schizothecium* plays a critical role in regulating SOM transformation and nitrogen cycling dynamics. Ma et al. (2022) demonstrated that long-term STR treatment enhanced the abundance of *Schizothecium*, improved the cycling and transformation of SOM, and consequently increased SOM content through microbial-mediated mechanisms, which ultimately contributes to improving wheat yield [66]. The *Schizothecium* secreted cellulase to decompose straw, so it was significantly enriched in the STR treatment [39]. Meanwhile, its proliferation inhibited the ammonia oxidation and reduced nitrogen leaching, thereby increasing total nitrogen content [82].

The *Vicinamibacteraceae* is a genus in the *Acidobacteria* phylum, and it can increase the PE by decomposing the refractory soil organic matter [83,84]. In addition, the abundance of *Vicinamibacteriaceae* was positively correlated with mineral-associated organic carbon content and regulated the accumulation and depletion of SOC [85]. Guo et al. (2025) found that *Vicinamibacteraceae* was the main bacterial genus with different nitrogen fertilizer levels and was positively correlated with the available N content in the soil [86]. In this study, the *Vicinamibacteraceae* were also identified as keystone species in the STR treatment. They increased the efficiency of organic matter decomposition [87]. Our study demonstrated that SOM, TN, and the C/N ratio were positively correlated with the relative abundance of *Vicinamibacteraceae*, which is in agreement with other researchers [88]. Yin et al. (2022) observed that the relative abundance of *Vicinamibacteraceae* increased significantly after adding corn straw biochar and was highly positively correlated with soil nutrient availability, improving crop growth and yield [89].

Moreover, our study found that crop yield was positively correlated with the relative abundance of *Schizothecium*, cellulose-degrading bacteria highly enriched in soils to which straw has been returned [71]. They convert lignin and cellulose into humus, and their abundance is significantly positively correlated with wheat yield [14,71]. In summary, the short-term STR and MF applications improve soil nutrients, microbial diversity, and soybean yield. However, the short-term application of STR may result in limitations for soil properties and microbial diversity due to incomplete decomposition into available nutrients, potentially leading to lower yields compared to long-term application of STR treatment. Additionally, the initial decomposition products from the additions of STR and MF may not provide the same level of stability as long-term treatment, thereby affecting soil fertility and microbial community composition, which require continuous monitoring in the future.

## 5. Conclusions

STR and MF significantly increased soil nutrients and improved the α diversity of soil microorganisms. Notably, the MF treatment increased the abundance and diversity of the soil’s microbial community during the crop growth season. Soybean yield was significantly positively correlated with the abundance of *Schizothecium* (the key fungal group in the STR treatment) and *Massilia* (the key bacterial group in the MF treatment). Notably, the STR and MF treatments increased soybean yield by 34.52% and 68.8%, respectively. The MF treatment substantially increased soil nutrient content and significantly enhanced the diversity indices in all sampling periods. In summary, these findings support the use of short-term organic inputs as an emergency soil restoration practice in degraded black soil regions, with implications for climate-resilient agriculture and microbial ecosystem engineering.

## Figures and Tables

**Table 1:** The initial soil properties of parent material.

TC (g/kg)	TN (g/kg)	TP (g/kg)	TK (g/kg)	Sand (%)	Slit (%)	Clay (%)	BD (g cm^−3^)	CEC cmol(+)/kg	NH_4_^+^-N (mg/kg)	NO_3_^−^-N (mg/kg)	AP (mg/kg)	pH
2.88	0.57	0.55	9.62	15.1	52.5	32.4	1.50	10.1	0.40	0.56	62.15	6.48

Note: TC, total carbon, TN, total nitrogen, TP, total phosphorus, TK, total potassium, Sand, sand content, Slit, soil porosity, Clay, clay content, BD, bulk density, CEC, cation exchange capacity, NH_4_^+^-N, ammonium nitrogen. NO_3_^−^-N, nitrate nitrogen, AP, available phosphorus.

**Table 2:** Soil physical and chemical properties of soil under different organic materials returned to the field.

Treatments	Indices						
	pH	ST (°C)	SWC (%)	TC (mg/kg)	TN (mg/kg)	C/N	SOM (mg/kg)
Seedling stage							
MF	6.90 ± 0.21 a	17.02 ± 1.13 a	13.76 ± 1.77 a	4350 ± 309 a	440 ± 50 a	9.88 ± 0.83 a	4450 ± 510 a
STR	6.37 ± 0.32 b	17.20 ± 1.19 a	12.81 ± 1.54 a	4000 ± 233 a	400 ± 50 ab	9.84 ± 0.52 a	4110 ± 440 b
CK	6.37 ± 0.20 b	16.98 ± 0.98 a	13.89 ± 1.01 a	3310 ± 204 b	340 ± 20 b	9.68 ± 0.31 a	3590 ± 280 c
Flowering stage							
MF	6.04 ± 0.23 a	29.25 ± 1.12 a	17.76 ± 3.07 a	6090 ± 518 a	820 ± 120 a	7.38 ± 0.93 a	5650 ± 570 a
STR	5.84 ± 0.34 a	29.00 ± 1.08 a	19.28 ± 4.20 a	6210 ± 1256 a	770 ± 90 a	7.83 ± 1.35 a	5750 ± 850 ab
CK	5.82 ± 0.22 a	28.59 ± 0.78 a	18.26 ± 2.12 a	3180 ± 238 b	690 ± 100 b	4.61 ± 0.11 b	3940 ± 450 b
Maturity stage							
MF	7.15 ± 0.14 a	18.14 ± 2.07 a	26.08 ± 2.42 a	6570 ± 1020 a	650 ± 120 a	10.21 ± 0.39 ab	6590 ± 1250 a
STR	6.12 ± 0.39 b	17.91 ± 2.33 a	25.58 ± 1.89 a	5560 ± 990 a	510 ± 60 ab	10.69 ± 0.67 a	5530 ± 440 ab
CK	6.52 ± 0.33 b	18.35 ± 3.22 a	26.38 ± 3.01 a	3710 ± 140 b	390 ± 20 b	9.54 ± 0.26 b	4680 ± 380 b
Mean values							
MF	6.70 ± 0.53 a	24.74 ± 5.10 a	19.05 ± 5.74 a	5670 ± 1190 a	640 ± 190 a	9.22 ± 1.44 a	5890 ± 1750 a
STR	6.11 ± 0.40 b	24.58 ± 5.11 a	19.70 ± 5.49 a	5240 ± 1340 a	570 ± 180 ab	9.52 ± 1.38 ab	5190 ± 950 ab
CK	6.19 ± 0.43 b	24.66 ± 4.94 a	18.76 ± 5.93 a	3410 ± 280 b	470 ± 150 b	7.89 ± 2.41 b	4040 ± 550 b

Note: CK: the control treatment without the additions of straw and manure fertilization; STR: Straw returning; MF: Manure fertilization. ST: soil temperature; SWC: soil moisture content; TC: soil total carbon; TN: soil total nitrogen; C/N: soil carbon-nitrogen ratio; SOM: soil organic matter. The different letters in each column represent significant differences among treatments (*p* < 0.05).

**Table 3:** Effects of different organic materials on topological properties of soil bacterial and fungal networks.

Network Metrics	Bacteria			Fungi		
	CK	STR	MF	CK	STR	MF
Nodes	145	148	134	88	99	73
Edges	1141	1386	502	169	197	96
Positive	73.62%	76.55%	82.27%	74.56%	84.26%	90.62%
Negative	26.38%	23.45%	17.73%	25.44%	15.74%	9.38%
Path length	2.777	2.683	3.875	3.734	3.678	3.989
Graph density	0.109	0.138	0.056	0.044	0.041	0.037
Diameter	7	7	11	9	10	9
Clustering coefficient	0.548	0.572	0.476	0.395	0.36	0.313
Average degree	15.738	19.521	7.493	3.841	3.98	2.63
Modularity	0.433	0.449	0.581	0.513	0.644	0.661

Note: CK: Degraded black soil in a natural state; STR: Straw returning; MF: Manure fertilization; Nodes represent the operation classification unit OTU, the nodes are classified by the phylum level of the door and scaled proportionally to the number of connections (node degrees). Draw lines where correlation coefficients are greater than 0.7 and *p* < 0.01. Path length: Shortest path between any two nodes. Graph density: the ratio of the number of edges to the total number of possible edges in a graph. Diameter: the longest and shortest path between any two nodes in the network. The clustering coefficient reflects the number of connections between the neighboring nodes. Average degree: the average number of connections of all nodes. Modularity: reflects the extent to which the network is divided into different modules.

## Data Availability

The original contributions presented in this study are included in the article/Supplementary Material. Further inquiries can be directed to the corresponding author.

## Supplementary Materials

The following supporting information can be downloaded at: https://www.mdpi.com/article/10.3390/microorganisms13051137/s1, Table S1: The fungal genus in straw return (STR) and manure fertilization (MF)treatments; Table S2: The bacterial genus in STR and MT treatments.
